# Association of Medicaid expansion and 1115 waivers for substance use disorders with hospital provision of opioid use disorder services: a cross sectional study

**DOI:** 10.1186/s12913-023-09035-0

**Published:** 2023-01-26

**Authors:** Ji Eun Chang, Cory E. Cronin, Zoe Lindenfeld, José A. Pagán, Berkeley Franz

**Affiliations:** 1 Department of Public Health Policy and Management, School of Global Public Health, New York University, 726 Broadway, New York, NY 10012 USA; 2 Heritage College of Osteopathic Medicine, Ohio University, 1 Ohio University, Athens, OH 45701 USA; 3 College of Health Sciences and Professions, Ohio University, 1 Ohio University, Athens, OH 45701 USA

**Keywords:** Medicaid, Substance-Related Disorders, Opioid-Related Disorders, Social Determinants of Health, Hospitals

## Abstract

**Introduction:**

Opioid-related hospitalizations have risen dramatically, placing hospitals at the frontlines of the opioid epidemic. Medicaid expansion and 1115 waivers for substance use disorders (SUDs) are two key policies aimed at expanding access to care, including opioid use disorder (OUD) services. Yet, little is known about the relationship between these policies and the availability of hospital based OUD programs. The aim of this study is to determine whether state Medicaid expansion and adoption of 1115 waivers for SUDs are associated with hospital provision of OUD programs.

**Methods:**

We conducted a cross-sectional study of a random sample of hospitals (*n* = 457) from the American Hospital Association’s 2015 American Hospital Directory, compiled with the most recent publicly available community health needs assessment (2015–2018).

**Results:**

Controlling for hospital characteristics, overdose burden, and socio-demographic characteristics, both Medicaid policies were associated with hospital adoption of several OUD programs. Hospitals in Medicaid expansion states had significantly higher odds of implementing any program related to SUDs (OR: 1.740; 95% CI: 1.032–2.934) as well as some specific activities such as programs for OUD treatment (OR: 1.955; 95% CI: 1.245–3.070) and efforts to address social determinants of health (OR: 6.787; 95% CI: 1.308–35.20). State 1115 waivers for SUDs were not significantly associated with any hospital-based SUD activities.

**Conclusions:**

Medicaid expansion was associated with several hospital programs for addressing OUD. The differential availability of hospital-based OUD programs may indicate an added layer of disadvantage for low-income patients with SUD living in non-expansion states.

**Supplementary Information:**

The online version contains supplementary material available at 10.1186/s12913-023-09035-0.

## Introduction

The last two decades have witnessed a large rise in opioid-related morbidity and mortality across the United States (U.S.). While the opioid epidemic has affected individuals across the socioeconomic spectrum, low-income individuals and people living in poverty are especially at risk for opioid addiction and overdose [1]. Lower-income individuals are more likely to have misused opioids or have an opioid use disorder (OUD) than the general population, and the risk of fatal overdose is significantly higher among people in low compared to high socio-economic status [2, 3]. As the nation’s primary social safety net insurer and as largest payer of substance use disorder (SUD) services in the U.S., Medicaid plays a critical role in shaping the delivery and reimbursement for OUD services across health care delivery settings [4].

Two Medicaid policies may be particularly salient. First, the 2010 Patient Protection and Affordable Care Act (ACA) allowed states to expand Medicaid eligibility to nonelderly adults with incomes up to 138 percent of the federal poverty level and required that individuals receiving coverage through the expansion be provided with mental health and SUD services on parity with other medical and surgical services. Medicaid expansion can increase access to SUD services and reduce the addiction-treatment gap in various ways. First, by expanding the pool of covered individuals eligible for treatment, Medicaid expansion can increase the demand for SUD services. Numerous studies have documented the dramatic increase in coverage for people with OUD in states that expanded Medicaid [5], and a corresponding increase in the use of Medicaid funds for OUD services [6]. Providers may respond to this demand by expanding access points to meet the growing demand.

Meanwhile, Sect. 1115 of the Social Security Act provided states an avenue to test new approaches in Medicaid that differ from what is required by federal statute. Waivers give states flexibility in how they design and operate their Medicaid program and can serve a broadly defined purpose or narrowly target specific populations [7]. In 2015, the Centers for Medicare and Medicaid Services (CMS) created a new Sect. 1115 waiver for behavioral health and SUDs, which specifically allowed states to pay for a wider range of residential and inpatient SUD services than previously allowed. While the waivers require the project to remain budget neutral, they have the potential to expand the breadth of and access to mental health and SUD services by increasing the types and number of services being offered for patients with SUD [8]. However, like Medicaid expansion, not all states have opted to apply for SUD-related 1115 waiver and both policies continue to be debated and discussed at the state and federal levels.

Considerable evidence suggests that both Medicaid expansion and SUD-related 1115 waivers may play a role in opening access points for OUD care, particularly in outpatient and residential settings. For example, residential treatment centers in states that expanded Medicaid were more likely to offer psychiatric treatment for SUD, and there was a 34% increase observed in residential treatment settings located in expansion states over the past decade [9]. Similarly, outpatient SUD providers in expansion states were more likely to offer psychiatric medications [9], and Medicaid expansion was associated with a 43 percent increase in Medicaid-reimbursed prescriptions for medications to treat SUDs [10]. Additionally, state implementation of a SUD-related Medicaid 1115 waiver was associated with an increase in the proportion of outpatient admissions for opioid treatment insured by Medicaid in the state, as well as an increase in the proportion of patients receiving medication for OUD (MOUD) in outpatient facilities [11, 12].

However, much less is known about the relationship between these Medicaid policies and the availability of hospital-based services for OUD. Opioid-related hospital use has risen dramatically, placing hospitals at the frontlines of the opioid epidemic [13]. Nearly half a million people with an opioid use disorder (OUD) are discharged from the hospital each year, and total hospital costs related to opioid overdoses have been estimated at $2 billion annually [14, 15]. Studies suggest that the vast majority of hospitals view OUD as a critical public health need in their communities [16]. Hospitals that identified OUD as a pressing issue are more likely to engage in activities to address opioid use, as are hospitals that employ medical home models, and hospitals with greater numbers of beds, academic medical center status, critical access status, religious affiliation, and public ownership [17]. However, no study to date has examined the association between state Medicaid policies and the availability of hospital-based OUD services.

Broader evaluations of the consequences of Medicaid expansion on hospitals have found that hospitals located in expansion states experienced higher Medicaid patient volume and revenue, lower rates of uncompensated care, and improved financial margins compared to hospitals located in non-expansion states [18–20]. By reducing the financial burden of uncompensated care, Medicaid expansion has the potential to free up hospital resources to engage in activities to address perceived public health needs in their communities, including opioid use [21]. Similarly, participation in 1115 waivers can provide needed flexibility for hospitals to offer a broader range of OUD services in its communities.

The aim of this study is to examine the association between state Medicaid policies and hospital provision of OUD programs. Nonprofit hospitals must show that they engage in activities that provide benefits for their communities to maintain their tax-exempt status. As a result, hospitals may be motivated to participate in evidence-based interventions to reduce the burden of OUD in their communities, including the direct provision of OUD treatment and partnerships with other community providers for opioid-related services. These services can include programs aimed at prevention, early intervention, and treatment services directly provided or indirectly supported by hospitals. Such hospital-based or initiated OUD services can lead to high treatment engagement as well as provide successful linkages to outpatient care [22–27]. These hospital-based OUD programs are likely to be more critical given the rise in opioid-related overdoses and hospitalizations following the onset of Covid-19. As the burden of opioid use among low-income individuals continues to grow and as policymakers consider further reforms to Medicaid, it is important to understand the relationship between these Medicaid policies and hospital responses to the opioid crisis in their communities.

## Methods

### Data and sample

Our dataset was constructed from multiple sources. Because the population of interest in this study is the universe of non-profit general hospitals across the U.S., the sample was created utilizing American Hospital Association (AHA)’s 2015 Annual Survey Database (*N* = 2,715) Stratifying by state, we included a random sample of 20% of all U.S. general nonprofit community hospitals within each US state, rounding up to the nearest whole number of hospitals to create our dataset (*n* = 613). To assess the extent to which our 20% random sample of US hospitals represents the entire population of hospitals, we compared our sample hospitals with the full population of nonprofit hospitals included in the AHA Annual Survey. We conducted t tests or chi2 tests to examine the following characteristics: bed size, system membership, teaching status, and hospitals’ urban/rural location. With the exception of hospital bed-size, our sample hospitals were not significantly different from the entire AHA hospital population. Our sample did have a slightly higher average bed-size (mean 236 beds in the study sample vs. 178 in the AHA population).

For each hospital in the sample, we compiled the most recent complete round of publicly available community health needs assessment (CHNA) and implementation strategy (IS) from 2015–2018. Because these documents are mandated to be publicly available under the ACA, we obtain the majority of these documents from hospital websites. In cases were these documents were not available online, a member of the research team contacted the hospitals for the documents. We excluded hospitals that did not provide their CHNA documents or implementation strategies (*n* = 109).

For hospitals that remained in our sample, we manually coded the CHNAs and IS documents to determine whether substance use was included among the top five needs (“prioritized”) in the CHNA and what strategies were proposed to addressed these needs in the IS. According to federal regulations, hospitals must identify community health needs and existing gaps in services in their CHNA and adopt implementation strategies to meet the community health needs identified (Crossley et al., 2016). Because these documents indicate a “plan” to address identified needs, they do not guarantee hospitals actually offer these programs. However, the IRS does require hospitals to provide information on the impact of the proposed programs in subsequent CHNAs, which may encourage actual implementation of the strategies listed in their plans. IS documents were coded for each of eight types of programs for addressing OUD: SUD treatment; primary care; emergency department services; risk/harm reduction and education; social determinants; community coalition approaches; prescriber guidelines; and policy advocacy (Table 1). All coding and analysis of CHNAs and IS documents occurred in 2018 and 2019 and followed the process described by Wickramatilake and colleagues [28].

**Table 1:** Implementation strategy categories to address opioid use disorder (OUD)

Category	Description	# and (%) hospitals that adopted the strategy
Any OUD strategy	At least one strategy in any of the below categories	307 (67.1%)
Treatment	Programs that increase access to formal OUD treatment services, including hospital inpatient services, residential treatment programs, and medications for OUD	124 (27.1%)
Primary care	Programs that increase referrals to primary care and help patients establish a medical home	132 (28.9%)
Prescriber guidelines	Programs that aim to improve opioid prescribing practices in adherence with CDC guidelines or monitor prescribing trends	29 (6.35%)
Targeted risk education and harm reduction	Programs that aim to educate target populations on OUD and related topics or mitigate harm from misuse	156 (34.1%)
Social determinants	Programs that address upstream determinants of OUD, including socioeconomic status, physical environment, housing, food, social support, and health care access (based on typology from Kaiser Family Foundation) (Artiga & Hinton, 2018)	32 (7.00%)
Emergency Department services	Programs that initiate treatment which are embedded in the ED	70 (15.3%)
Policy advocacy	Programs that support policy change at the institutional, local, state, or federal level	21 (4.60%)
Community coalition building	Programs that facilitate collaboration with key stakeholders to address OUD at the community level	115 (25.2%)

Table 1 describes strategies to address opioid use disorder by hospitals coded from the community health needs assessments, and the number and percentage of hospitals from our sample (*n* = 457) that adopted each strategy

Our key predictor variables of interest were binary measures indicating whether the state expanded Medicaid and whether the state obtained a Medicaid 1115 waiver for SUD funds. Both variables were obtained from the Kaiser Family Foundation, which provided the year in which each policy was adopted. Separate indicator variables were created to identify whether each policy was adopted in the year the hospital submitted their CHNA (ranging from 2015–2018) [29] (see Additional file 1: Appendix Table 1).

We compiled additional variables from secondary data sources in order to control for organizational characteristics and broader community-level factors that may influence hospital adoption of OUD-related services. Hospital characteristics were sourced from the 2015 AHA Annual Survey and included teaching hospital status (yes/no), system membership status (yes/no), and hospital size (< 400 beds or 400 + beds). We also included a variable indicating whether each hospital is designated as a safety net hospital (yes/no), based on membership in America's Essential Hospitals or designation as a critical access hospital [30, 31]. County characteristics included the percent of residents in the county who are White, the rate of preventable hospitalizations in the county (per 100,000 Medicare enrollees), sourced from County Health Rankings, as well as data on county poverty rates (percent of residents at or under 138% FPL), uninsured rates, and urban/rural designation sourced from the Area Health Resource File [32]. From the Centers for Disease Control and Prevention, we used a variable indicating the county rate for fatal drug overdoses per 100,000 residents [33, 34]. To control for the broader policy environment surrounding OUD use, we obtained a state-level variable on the legality of syringe exchange from amFAR.org [35]. Finally, states can shape what treatment options patients have access to by determining coverage for MOUD, widely considered the gold-standard for OUD treatment. State coverage of MOUD was based on responses to the 2015 National Association of State Alcohol and Drug Abuse Directors questionnaire [24]. After accounting for missing data, the analytic sample for the study included 457 hospitals across 49 states (see Additional file 1: Appendix Table 1).

### Analytic strategy

We mapped the presence of the two state Medicaid policies related to OUD and how these policies tended to overlap using data visualization software Tableau. In bivariate analyses, we used logistic regression to examine the associations between the Medicaid policies and hospital adoption of the eight types of programs to address OUD, as well as whether substance use was included in the implementation strategy. In multivariable analyses, we added hospital, county, and state-level control variables to the logistic regression models to examine the relation between the two policies and hospital adoption of OUD strategies. Given the nesting of hospitals within counties and counties within states in our dataset, we used a two-level model (first level hospital, second level state) for multivariable models. Statistical analyses were 2-tailed and conducted using Stata SE16.1. (Statacorp) [36] Significance was established throughout at *P* < 0.05.

## Results

Table 1 shows that approximately two-thirds (67.2%) of hospitals adopted at least one opioid-related program in their implementation strategy and that the three most common programs were targeted risk education and harm reduction (34.1%), formal treatment (27.1%), and primary care (28.9%).

**Fig. 1:**
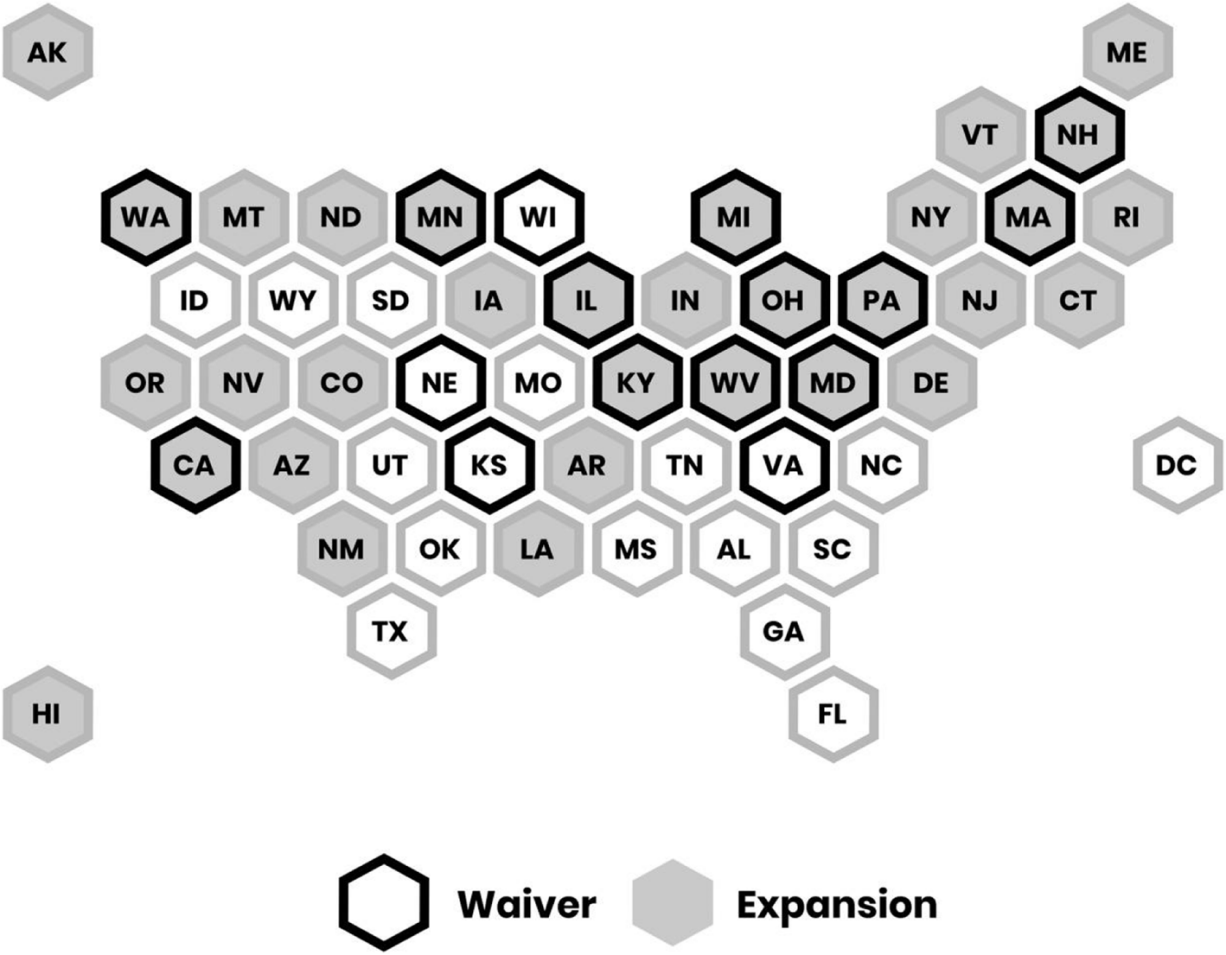
State adoption of OUD-related Medicaid policies (2015–2018)

Approximately two-thirds of hospitals (68%) were in a state that had expanded Medicaid prior to the publication of their CHNA, and about half (51.42%) were in states that had a 1115 Waiver for SUD treatment in place (Table 2). See Fig. 1 for a geographic distribution of state Medicaid programs and policies. About two-fifths (39.61%) of the hospitals in our sample were located in states that both expanded Medicaid and adopted 1115 SUD waivers, while a fifth (19.69%) were in states that adopted neither.

**Table 2:** Descriptive statistics

*Variable*	*Mean*
State Medicaid Policies	
Medicaid Expansion*	68.49%
1115 Waiver for SUD Treatment*	51.42%
Both Expansion and 1115	39.61%
None	19.69%
Hospital Characteristics	
Greater than 400 beds	15.32%
Academic Medical Center	8.75%
Hospital in System	71.77%
Safety-net Hospital	27.35%
County Characteristics	
% Under Poverty	22.44%
% Uninsured Adults	18.05%
Overdose Rate	15.38 per 100,000
Preventable Hospital Stays	22.70 per 100,000
% White residents	73.26%
State OUD Policies	
State with Legal SSP	52.44%
MOUD Funding	52.44%

^*^ as of the year the CHNA was submitted (2015–2018)

Only a small proportion (15.3%) of hospitals in our sample were large (over 400 beds) or part of an academic institution (8.75%). About three quarters of our sample were part of a larger hospital system, and approximately 27% of sample hospitals were safety net institutions. More than 1 in 5 residents (22%) were below the poverty line in the counties served by our sample’s hospitals and slightly less than 1 in 5 adults (18%) were uninsured in these counties. The average overdose rate for the included counties was 15.38 for every 100,000 residents and the percentage of county residents that were White was 73%. About three quarters of the hospitals (73%) were in states that allow syringe exchange programs to operate while slightly more than half (52%) of hospitals were located in states that had Medicaid funding for medications to treat OUD specifically.

In unadjusted analyses, we found that both Medicaid policy variables were significantly and positively associated with hospital reporting plans to adopt any OUD programs (Table 3). Both policy variables were also positively and significantly associated with hospital plans to adopt specific programs, though the impact varied. Specifically, hospitals located in states that expanded Medicaid had significantly higher odds of planning to adopt programs for treatment (OR: 2.347; 95% CI: 1.426–3.865) and social determinants (OR: 7.527; 95% CI: 1.773–31.94). Meanwhile, hospitals in states that participated in the 1115 SUD waivers were significantly more likely to plan to offer primary care-based programs (OR: 1.764; 95% CI: 1.167–2.664) and ER services (OR: 1.866; 95% CI: 1.101–3.164).

**Table 3:** Unadjusted logistic regression estimating association between medicaid policies and hospital adoption of OUD strategies (*n* = 457)

OUD Strategy	Medicaid Expansion OR	1115 SUD Waiver OR
Prioritized SUD	2.411** (1.596—3.643)	1.690** (1.139—2.506)
Substance Abuse Treatment	2.347** (1.426—3.865)	1.262 (0.834—1.910)
Primary Care	1.399 (0.892—2.193)	1.764** (1.167—2.664)
Prescriber Guidelines	1.824 (0.726—4.582)	0.649 (0.303—1.392)
Risk Ed and Harm Reduction	1.531 (0.995—2.355)	1.356 (0.919—2.000)
Social Determinants	7.527** (1.773—31.94)	1.076 (0.524—2.211)
ER Services	1.833 (0.997—3.370)	1.866* (1.101—3.164)
Policy	1.498 (0.538—4.171)	1.274 (0.526—3.084)
Community Coalition	1.069 (0.677—1.690)	1.255 (0.821—1.919)

Confidence intervals in parenthesis

^**^ *p* < 0.01

^*^p < 0.05

In multivariate models, we found that states that expanded Medicaid had significantly higher odds of planning to implement any program related to SUDs (OR: 1.740; 95% CI: 1.032–2.934) as well as specific programs to provide OUD treatment (OR: 1.955; 95% CI: 1.245–3.070) and address the social determinants of substance use (OR: 6.787; 95% CI: 1.308–35.20), even after controlling for key institutional, socio-demographic, and policy factors (Table 4). However, State 1115 waivers to support SUD treatment were not significantly associated with the planned adoption of any hospital-based SUD programs.

**Table 4:** Adjusted logistic regression estimating association between Medicaid policies and hospital adoption of OUD strategies (*n* = 457)

**VARIABLE**	**Prioritized SUD** OR	**Substance Abuse Treatment** OR	**Primary Care** OR	**Prescriber Guidelines** OR	**Risk Ed and Harm Reduction** OR	**Social Determinants** OR	**ER Services** OR	**Policy** OR	**Community Coalition** OR
***State Medicaid Policies***									
**Medicaid Expansion**	1.740** (1.032—2.934)	1.955** (1.245—3.070)	0.683 (0.266—1.754)	1.133 (0.463 – 2.774)	1.290 (0.708—2.352)	6.787** (1.308—35.20)	1.668 (0.766—3.634)	0.919 (0.288—2.935)	0.914 (0.425—1.968)
**1115 Waiver for SUD Treatment**	1.409 (0.869—2.286)	1.079 (0.713—1.633)	2.003 (0.917 – 4.375)	0.546 (0.252—1.184)	1.278 (0.728—2.245)	0.735 (0.312—1.733)	1.788 (0.763 – 4.191)	1.543 (0.465 – 5.122)	1.360 (0.632—2.925)
***Hospital Characteristics***									
**Greater than 400 Beds**	1.247 (0.696—2.235)	2.158* (1.013—4.599)	0.531 (0.244—1.154)	1.302 (0.399—4.254)	1.245 (0.626—2.476)	0.467 (0.0842 – 2.592)	2.057 (0.809 – 5.232)	1.750 (0.453—6.756)	0.791 (0.395—1.582)
**Academic Medical Center**	0.974 (0.406—2.339)	1.091 (0.323—3.687)	2.111 (0.954 – 4.673)	4.195 (0.949 – 18.54)	0.939 (0.321—2.749)	0.825 (0.0691 – 9.852)	0.569 (0.117 -2.769)	1.827 (0.208 – 16.02)	0.571 (0.185—1.761)
**Hospital in System**	1.197 (0.779—1.959)	1.010 (0.640—1.594)	1.089 (0.692—1.715)	0.900 (0.403—2.009)	1.684 (0.877 – 3.236)	1.964 (0.625 – 6.167)	0.614 (0.356—1.058)	1.689 (0.387 – 7.377)	1.263 (0.684 – 2.334)
**Safety-net Hospital**	0.956 (0.652—1.403)	1.425 (0.828 – 2.453)	0.918 (0.503—1.677)	0.549 (0.165—1.826)	1.093 (0.614—1.945)	1.735 (0.579—5.198)	1.123 (0.531—2.371)	0.456 (0.070 – 2.942)	1.409 (0.822—2.415)
***County Characteristics***									
**% Under Poverty**	1.003 (0.962—1.046)	0.994 (0.945—1.046)	1.011 (0.942—1.086)	1.056 (0.984—1.133)	0.975 (0.933—1.018)	0.972 (0.898—1.053)	1.052 (0.990—1.119)	1.006 (0.883—1.146)	1.037 (0.991—1.084)
**% Uninsured Adults**	0.979 (0.936—1.025)	0.952 (0.906—1.001)	0.964 (0.902—1.030)	0.918* (0.847—0.994)	1.008 (0.953—1.065)	1.022 (0.934—1.119)	0.969 (0.904—1.039)	0.973 (0.887—1.068)	0.964 (0.903—1.029)
**Overdose Rate**	1.033 (0.994—1.074)	1.055** (1.0616—1.096)	1.034 (0.986—1.085)	1.001 (0.938—1.069)	0.999 (0.955—1.046)	1.042 (0.987—1.101)	0.958 (0.899—1.021)	1.044 (0.944—1.155)	1.027 (0.985—1.071)
**Preventable Hospital Stays**	0.985** (0.9747—0.993)	0.983* (0.968—0.998)	0.982 (0.964 – 1.000)	1.002 (0.982—1.022)	1.003 (0.990—1.015)	0.987 (0.964—1.011)	0.989 (0.976—1.003)	0.981 (0.954—1.009)	0.983 (0.961 – 1.006)
**% White residents**	1.013 (0.999—1.027)	1.015 (0.994—1.036)	1.001 (0.977—1.025)	1.047** (1.013—1.081)	1.019** (1.005—1.033)	0.990 (0.960—1.021)	1.003 (0.9886- 1.020)	1.018 (0.990—1.046)	1.014 (0.998—1.029)
***State OUD Policies***									
**State with Legal SSP**	1.533 (0.882—2.663)	0.986 (0.588—1.653)	2.380 (0.976 – 5.804)	1.787 (0.822 – 3.884)	1.518 (0.891—2.584)	1.556 (0.530—4.567)	1.000 (0.412 – 2.431)	2.499 (0.841—7.420)	1.659 (0.676 – 4.073)
**MOUD Funding**	1.915* (1.095—3.350)	1.471 (0.932—2.323)	0.857 (0.401—1.829)	0.896 (0.367—2.188)	1.171 (0.670 – 2.047)	1.770 (0.723 – 4.337)	2.249* (1.002 – 5.045)	0.597 (0.162 – 2.192)	1.245 (0.596—2.602)

^**^ *p* < 0.01

^*^ *p* < 0.05

## Discussion

In this study, we found that Medicaid expansion was associated with some, though not all, hospital-based planned programs for addressing OUD. Specifically, hospitals in Medicaid expansion states had six times the odds of including programs that address the social determinants of health (SDOH) in their implementation strategy than counterparts in non-expansion states, even after controlling for hospital characteristics, overdose burden, and socio-demographic characteristics. SDOH describes the range of social, environmental, and economic factors that often play a disproportionately large role in health outcomes for vulnerable populations including low-income individuals and people with SUDs. Medicaid programs are uniquely placed to support programs targeting SDOH given their central role in supporting low-income Americans. Prior studies found that state Medicaid programs are increasingly interested in integrating services that address SDOH into program design but concerns about sustainable funding looms [37, 38]. Prior studies documented lower insurance coverage among patients with SUD in states that did not expand Medicaid. The differential availability of hospital-based OUD programs may indicate an added layer of disadvantage for low-income patients with SUD living in non-expansion states.

A large body of evidence suggests hospital-based strategies are effective and necessary to address the growing opioid crisis [17–23]. Yet a third of hospitals in our sample did not report any planned program to address opioid use in their community, and most planned strategies were listed by only a small minority of hospitals. Recent studies point toward capacity constraints and lack of funding as primary drivers of hospital decisions not to adopt OUD-related programs [33]. Even when CHNAs indicate that responding to opioid use would be important to hospitals’ communities, they may not feel equipped to implement key strategies without the resources made available to hospitals through supportive policies. Hospitals may feel better prepared to undertake such strategies when supported by state policies such as Medicaid Expansion and Sect. 1115 waivers that enable greater flexibility and funding for OUD-related services. As of July 2021, more than a decade after the passage of the ACA, 12 states have not adopted the expansion of Medicaid. Similarly, 17 states do not have Sect. 1115 waivers for SUD treatment [29]. The American Rescue Plan Act, the COVID-19 relief package that became law in March 2021, included a number of provisions to encourage states that have not yet adopted Medicaid expansion to do so. The fact that hospitals are providing high yield services in states that have expanded Medicaid and have specific 1115 waivers targeting SUDs suggest that states who have been affected greatly by the U.S. opioid epidemic may have much to gain from adopting these policy approaches.

Our data and findings have important limitations to consider. First, we relied on community benefit documents to assess hospital-based services which by definition limits our focus to non-profit hospitals which account for approximately two-thirds of hospitals nationally. Second, hospitals report only an “implementation strategy” which lists programs they plan to offer. As such, it is not possible to assess whether these programs were actually implemented or their effectiveness. Third, we utilized a cross-sectional approach which provides data on programs offered during the first and only full round of the community benefit reporting cycle (2015–2018). Our cross-sectional approach only assesses associations and does not allow us to interpret a causal relationship across variables. Future studies will be necessary to assess trends in services over time using the next round of reports (2019–2021) and how hospitals addressed OUD in the context of the COVID-19 pandemic.

Other limitations include that while the ACA required state Medicaid programs to cover SUD treatment for their Medicaid expansion population, it allowed states to decide which individual services are reimbursable. Though our multivariate model controlled for Medicaid funding for MOUD, it did not capture state-level differences in coverage for other SUD and OUD treatment [5]. Similarly, our explanatory variable did not capture nuanced differences in the design of 1115 waivers across states, nor did it capture the full spectrum of organizational or county-level factors that may influence hospital decisions to offer OUD services, hence resulting in omitted variable bias. Furthermore, the definition of OUD programs in our paper includes a specific set of OUD strategies that may or may not align with what may have changed in a 1115 waiver. For example, hospitals may have been incentivized to add inpatient and residential services from an 1115 waiver – this effort would not be captured in the categories coded in our paper. Finally, while we included a state-level variable on the legality of syringe exchange as a proxy for different cultural or policy orientations that shape willingness to offer OUD services, it is possible that states who adopted various Medicaid programs were also similar in other ways that were not accounted for in our models.

## Conclusion

The opioid epidemic is an ongoing public health crisis that requires sustained, coordinated efforts from across the healthcare delivery system including hospitals. Hospital responses to this crisis have become particularly relevant as the rate of opioid-related hospitalizations surged amidst the COVID-19 pandemic, especially among low-income adults with SUD. When considering responses to OUD, our findings point toward a link between state policies and the specific strategies put forward by hospitals. Policymakers and state leaders who wish to engage hospitals further in efforts to address issues of opioid use in their communities should be aware of this relationship.

## Supplementary Information

**Additional file 1:** **Appendix Table 1. **Sample state policy characteristics.

## Data Availability

The dataset compiled and analyzed during the current study are available from the corresponding author on reasonable request. The hospital CHNAs and implementation strategies are publicly available on hospital websites. Data on our key policy predictors were obtained from https://www.kff.org/medicaid/issue-brief/medicaid-waiver-tracker-approved-and-pending-section-1115-waivers-by-state/ (publicly available). Data on hospital characteristics are available from https://essentialhospitals.org/about-americas-essential-hospitals/listing-of-americas-essential-hospitals-members (publicly available) and https://www.ruralhealthinfo.org/topics/critical-access-hospitals (publicly available). Data on county-level characteristics are available from https://datawarehouse.hrsa.gov/topics/ahrf.aspx (publicly available). Data on county-level overdose rates are available from the CDC: https://www.cdc.gov/drugoverdose/data/analysis.html (publicly available). Data on state MOUD coverage was obtained from https://opioid.amfar.org/indicator/SSP_legality (publicly available).

## Abbreviations

SUDs: Substance use disorders
OUD: Opioid use disorder
U.S: United States
ACA: 2010 Patient Protection and Affordable Care Act
CMS: Center for Medicare and Medicaid Services
MOUD: Medication for Opioid Use Disorder
AHA: American Hospital Association
CHNA: Community Health Needs Assessment
