# Supplementary material for: Association of Medicaid expansion and 1115 waivers for substance use disorders with hospital provision of opioid use disorder services: a cross sectional study
Source: BMC Health Serv Res. 2023 Jan 26;23:87. doi: 10.1186/s12913-023-09035-0 (PMC9877490; doi:10.1186/s12913-023-09035-0)
Supplement: Supplementary file 1 — Additional file 1: Appendix Table 1. Sample state policy characteristics. [file 12913_2023_9035_MOESM1_ESM.docx]

**Appendix Table 1:** Sample State Policy Characteristics

| **State** | **Number of Hospitals per State** | **Medicaid Expansion*** | **1115 Waiver for SUD**** |
| --- | --- | --- | --- |
| AK | 1 | Yes | No |
| AL | 2 | No | No |
| AR | 4 | Yes | No |
| AZ | 4 | Yes | No |
| CA | 34 | Yes | Yes |
| CO | 6 | Yes | No |
| CT | 4 | Yes | No |
| DE | 1 | Yes | No |
| FL | 6 | No | No |
| GA | 9 | No | No |
| HI | 2 | Yes | No |
| IA | 7 | Yes | No |
| ID | 3 | No | No |
| IL | 23 | Yes | Yes |
| IN | 9 | Yes | No |
| KS | 6 | No | Yes |
| KY | 10 | Yes | Yes |
| LA | 4 | Yes | No |
| MA | 11 | Yes | Yes |
| MD | 6 | Yes | Yes |
| ME | 8 | Yes | No |
| MI | 20 | Yes | Yes |
| MN | 19 | Yes | Yes |
| MO | 12 | No | No |
| MS | 6 | No | No |
| MT | 9 | Yes | No |
| NC | 11 | No | No |
| ND | 9 | Yes | No |
| NE | 9 | No | Yes |
| NH | 5 | Yes | Yes |
| NJ | 9 | Yes | No |
| NM | 3 | Yes | No |
| NV | 1 | Yes | No |
| NY | 35 | Yes | No |
| OH | 22 | Yes | Yes |
| OK | 5 | No | No |
| OR | 10 | Yes | No |
| PA | 18 | Yes | Yes |
| RI | 2 | Yes | No |
| SC | 4 | No | No |
| SD | 6 | No | No |
| TN | 17 | No | No |
| TX | 16 | No | No |
| UT | 4 | No | No |
| VA | 13 | No | Yes |
| VT | 4 | Yes | No |
| WA | 8 | Yes | Yes |
| WI | 26 | No | Yes |
| WV | 5 | Yes | Yes |
| TOTAL: | 468 | 32 | 16 |

*States that expanded Medicaid prior to the submission date of last CHNA

**States that obtained an 1115 waiver for SUD prior to the submission date of last CHNA
